# Can the Reboot coaching programme support critical care nurses in coping with stressful clinical events? A mixed-methods evaluation assessing resilience, burnout, depression and turnover intentions

**DOI:** 10.1186/s12913-023-10468-w

**Published:** 2024-03-15

**Authors:** K. S. Vogt, J. Johnson, R. Coleman, R. Simms-Ellis, R. Harrison, N. Shearman, J. Marran, L. Budworth, C. Horsfield, R. Lawton, A. Grange

**Affiliations:** 1 Bradford Institute for Health Research, Bradford Royal Infirmary, Temple Bank House, Duckworth Lane, Bradford, BD9 6RJ UK; 2 Department of Psychology, University of Leeds, Leeds, LS2 9JT UK; 3 School of Population Health, University of New South Wales, Sydney, 2052 Australia; 4 Centre for Health Systems and Safety Research: Australian Institute of Health Innovation, Macquarie University, Sydney, Australia; 5 Leeds Teaching Hospitals NHS Trust, Great George Street, Leeds, LS1 3EX UK; 6 Leeds Institute of Rheumatic and Musculoskeletal Medicine, University of Leeds, Leeds, UK; 7 School of Health and Wellbeing: College of Medical, Veterinary and Life Sciences, University of Glasgow, Clarice Pears Building, Glasgow, G12 8TB UK; 8 Department of Primary Care & Mental Health, Institute of Population Health, University of Liverpool, Eleanor Rathbone Building, Liverpool, L69 7ZA UK; 9 West Yorkshire Adult Critical Care Network, Leeds Teaching Hospitals, Leeds, UK; 10 NIHR Yorkshire & Humber Patient Safety Research Collaboration, Bradford Teaching Hospitals Foundation Trust, Bradford, UK; 11 Mid Yorkshire Teaching NHS Trust, Wakefield, UK

**Keywords:** Nurses, Critical care, Resilience, Burnout, Healthcare staff, COVID-19, Coaching, Intention to leave

## Abstract

**Background:**

Critical care nurses (CCNs) are routinely exposed to highly stressful situations, and at high-risk of suffering from work-related stress and developing burnout. Thus, supporting CCN wellbeing is crucial. One approach for delivering this support is by preparing CCNs for situations they may encounter, drawing on evidence-based techniques to strengthen psychological coping strategies. The current study tailored a Resilience-boosting psychological coaching programme [Reboot] to CCNs. Other healthcare staff receiving Reboot have reported improvements in confidence in coping with stressful clinical events and increased psychological resilience. The current study tailored Reboot for online, remote delivery to CCNs (as it had not previously been delivered to nurses, or in remote format), to (1) assess the feasibility of delivering Reboot remotely, and to (2) provide a preliminary assessment of whether Reboot could increase resilience, confidence in coping with adverse events and burnout.

**Methods:**

A single-arm mixed-methods (questionnaires, interviews) before-after feasibility study design was used. Feasibility was measured via demand, recruitment, and retention (recruitment goal: 80 CCNs, retention goal: 70% of recruited CCNs). Potential efficacy was measured via questionnaires at five timepoints; measures included confidence in coping with adverse events (Confidence scale), Resilience (Brief Resilience Scale), depression (PHQ-9) and burnout (Oldenburg-Burnout-Inventory). Intention to leave (current role, nursing more generally) was measured post-intervention. Interviews were analysed using Reflexive Thematic Analysis.

**Results:**

Results suggest that delivering Reboot remotely is feasible and acceptable. Seventy-seven nurses were recruited, 81% of whom completed the 8-week intervention. Thus, the retention rate was over 10% higher than the target. Regarding preliminary efficacy, follow-up measures showed significant increases in resilience, confidence in coping with adverse events and reductions in depression, burnout, and intention to leave. Qualitative analysis suggested that CCNs found the psychological techniques helpful and particularly valued practical exercises that could be translated into everyday practice.

**Conclusion:**

This study demonstrates the feasibility of remote delivery of Reboot and potential efficacy for CCNs. Results are limited due to the single-arm feasibility design; thus, a larger trial with a control group is needed.

**Supplementary Information:**

The online version contains supplementary material available at 10.1186/s12913-023-10468-w.

## Background

The healthcare professions are seen as some of the most stressful occupations, due to the close human contact, involvement with illness, death and dying, quick decision-making, risk of making errors and the involvement in adverse events they entail [1–6]. This stress and the demands on health care professionals (HCPs) have been exacerbated by the Covid-19 pandemic. Over the past 3 years, HCPs have had to cope with extreme emotional and physical stress, which has included redeployment, insufficient provision of medical supplies and personal protective equipment (PPE) and witnessing a record number of deaths among patients and colleagues. They have also been under pressure to adhere to ever-evolving infection control measures and have experienced anxiety about their personal health (as well as that of their families) [7–16]. Out of all areas of healthcare, Critical Care has been the most significantly affected clinical area by Covid-19 [17–19]. This has had detrimental effects to the psychological wellbeing of staff working in critical care units and is especially true for critical care nurses (CCNs) [19–26].

The international literature has consistently identified CCNs as having the worst outcomes on psychological wellbeing measures, such as depression, burnout, and post- traumatic stress disorder (PTSD) both during, and since the pandemic, and both compared to other critical care HCPs, such as physicians, and compared to non-critical care HCPs [21]. Two studies that illustrate the impact of working as a critical care nurse during the pandemic were conducted by Greenberg et al. [22] and Moll et al. [21]. In the United Kingdom (UK), Greenberg et al. surveyed 709 HCPs working in Critical Care on nine intensive care units (ICUs). Out of the three groups (doctors, nurses and *‘other’*), CCNs (*n* = 344; 49% of the sample) were *significantly more* likely to screen positive for depression (moderate, and severe), PTSD and anxiety (moderate, and severe). Further,19% of these nurses reported suicidal ideation [21]. In the US, Moll et al. compared the burnout scores of healthcare professionals working on critical care units between 2017 (*n* = 572, nurses *n* = 323) and 2020 (*n* = 710, nurses *n* = 372). Nurses were found to have the sharpest increase in burnout, despite increases in burnout across all professions surveyed. Taken together, these findings show that CCN wellbeing has been significantly impacted by the pandemic. Therefore, it essential that HCPs are supported in their wellbeing, and that they can draw on evidence-based techniques to recover from stressful events, without suffering negative psychological consequences.

Furthermore, poor CCNs wellbeing, the development of burnout and PTSD have been linked with intention to leave critical care nursing, and nursing altogether [15, 23, 27–29]. Thus, supporting CCNs’ wellbeing is not only a priority at an individual level (for individual CCNs), but must also be a priority at organizational level, to avoid further staff shortages [20]. One of the protective factors against the development of PTSD and burnout is psychological resilience [30–33]. Resilience refers to someone’s ability to maintain an emotional equilibrium during difficult experiences [34]. There is now increasing evidence that resilience can be increased with the help of psychological interventions [35, 36]. The Recovery-boosting [“Reboot”] coaching programme evaluated in the current study seeks to enhance HCP resilience by providing them with evidence-based psychological tools to prepare and recover from stressful clinical events. Reboot aims to develop psychological constructs known to confer resilience, including higher self-esteem, greater mental flexibility, and a more positive explanatory style for negative events [19, 37, 38]. Reboot was first developed and piloted in 66 HCPs and healthcare students; groups included paediatric doctors, midwives, and physician associate students [37]. It consisted of one 4-hour workshop and one 1-hour coaching phone call. At follow-up, participants showed significantly higher levels of psychological resilience and confidence in coping with adverse events; suggesting the intervention was feasible and acceptable to participants and potentially effective for increasing resilience. Although these results were promising, nurses were not included in the study [37]. Therefore, the feasibility of Reboot for nurses remains to be established through further research [19].

The COVID-19 pandemic generated an increased need for psychological support for nurses, particularly CCNs, given their significant distress and worse psychological outcomes than other critical care professionals [22, 25]. However, the pandemic also drastically reduced the feasibility of delivering in-person psychological interventions. Thus, the current study aimed to adapt the pre-existing Reboot programme for remote delivery for CCNs [19].

The *primary* objective was to assess the feasibility of delivering Reboot via online, remote delivery to CCNs. This was measured via demand, recruitment, and programme retention statistics.

The *secondary* objective was to provide a preliminary assessment of whether Reboot was associated with increases in self-reported psychological resilience and confidence in coping with adverse events, and decreases in depression and burnout, via analysis of questionnaires and interviews.

## Methods

A more detailed report of the methods can be found in the open-access study protocol paper [19].

### Study design & settings

A single-arm before-after feasibility study design was used; with a mixed-methods evaluation. Participants were invited to complete online questionnaires at five time points, which were Baseline (Time 1), following completion of two group workshops (Time 2), following completion of two individual coaching calls (Time 3), at 2-week follow-up post the final coaching call (Time 4). A fifth timepoint (Time 5) was added in May 2022 to investigate participants’ intention to leave nursing.

Online interviews were conducted with 25% of participants [who were randomly selected], after completion of the intervention. The Kirkpatrick model for assessing training interventions [39] was used, and four levels of outcome data were collected (Reaction, Learning, Behaviour, Results), as per Johnson et al. [37].

### Ethics approval

Ethics approval was granted by the School of Psychology, University of Leeds Ethics committee (approved on 25-08-2021, PSYC-302; an ethics amendment was approved on 09-05-2022, PSYC-535). The study adheres to both the British Psychological Society’s Code of Ethics and Conduct, as well as Declaration of Helsinki.

### Adaptation to online, remote delivery

Reboot was adapted for online delivery from a previous in-person group delivery method. This is reported in-depth in the study protocol [19]. The original intervention consisted of one 4-hour in-person group workshop and one 1-hour individual coaching phone call; and was delivered by a Clinical Psychologist (JJ) and an Occupational Health Psychologist (RSE) [37]. Adaptation for online, remote delivery involved changing this to two 2-hour online group workshops hosted via Zoom (each pair of workshops was termed a ‘cycle’), and two 1-hour individual coaching calls and was delivered by a Cognitive-behavioural (CBT) therapist (RC).

### Participants

The recruitment target was 80 CCNs working in the National Health Service (NHS) in the UK. Full inclusion/exclusion criteria, and sample size justification can be found in the protocol paper [19].

### Outcomes

#### Primary feasibility outcomes

As per protocol, feasibility outcomes were measured via demand [how many CCNs signed up], recruitment [how many CCNs consented and attended the first workshop], and retention [a) how many participants completed both workshops, b) how many participants completed both workshops and coaching calls, and c) how many completed the final follow-up questionnaires]. Using results of the in-person version of Reboot delivered by, feasibility success for the current study was met, if the following criteria were met:

- at least 80 CCNs signed up to the study (demand)
- at least 80 CCNs consented to taking part in the study and attend the first workshop (recruitment).
- at least 90% of recruited CCNs complete both workshops
- at least 70% completed both workshops and coaching calls, and
- at least 50% of recruited CCNs complete all follow-up measures, up to Time 4.

### Secondary outcomes

Secondary outcomes were resilience [measured via the *Brief Resilience Scale* (BRS)] [40]], confidence in coping with adverse events [measured via *Confidence in Coping with Adverse Events Questionnaire* [37]]*,* knowledge of resilience [measured via *Knowledge Assessment* [37]*, Burnout [*measured via an abbreviated version of the *Oldenburg Burnout Inventory (OLBI)* [41]], and depression [measured via the *Patient Health Questionnaire (PHQ-9)* [42]]. Feedback and reactions to the Reboot workshops [assessed via “Feedback” questionnaire [37] were also assessed. Internal reliability coefficients for the measures are reported in the ‘Results’ section of this paper.

#### Intention to leave

In addition to the above outcomes, an amendment was made to the original protocol to include a measure of intention to leave. All participants who completed the programme (both workshops, both coaching calls) were asked to answer an extra questionnaire as part of an additional follow-up survey. Participants were firstly asked to answer a set of questions measuring their turnover intentions as they recalled them prior to participating in Reboot (“*Think back to two weeks before you attended your first Reboot workshop, how were you feeling …?”* and then a set of questions about their current turnover intentions (“How are you feeling now …?”). More specifically, intention to leave was measured via three items for the two time points *(“I was/am planning to leave critical care nursing for another type of nursing”,* “*I was/am planning to leave nursing altogether”* and “*I was/am planning to continue working as a critical care nurse*” [reverse coded]) and answered on a scale from Strongly agree (1) to strongly disagree (5), with lower scores indicating lower intention to leave in critical care nursing.

### Procedure

Study information was circulated to the CCNs via Critical Care Networks and social media, via flyers, tweets, websites, and emails. A QR code could be used to access a website containing study information and a sign-up link. During sign-up, participants provided their details, and selected dates for workshops 1 and 2 from a list of cycles. Confirmation of dates was confirmed by email. Seven days prior to workshop 1, participants received an email with a questionnaire link, containing 1) consent form, 2) baseline survey, 3) a video to watch prior to attending the first workshop and 4) online links to access their workshops. Around the same time, participants also received a booklet in the post to use in the workshops, as well as a welcome phone call from the therapist facilitating the workshops. Both workshops took place via Zoom. At the end of the second workshop, the therapist asked participants to complete the Time-2 questionnaire and booked participants in for their two coaching calls. The coaching calls took place via phone or video call, depending on preference. After coaching call 2, participants completed Time-3 questionnaires, which they were sent by the therapist. Two to three weeks after the second coaching call, participants were emailed Time-4 questionnaires, and were invited to take part in an interview if selected (see Appendix 1 for Interview Guide). Interviewees were selected via random number generation from 0 to 100, numbers were assigned to participants in order of sign-up.

In May 2022, participants received a further questionnaire, assessing intention to leave critical care nursing. This questionnaire was an amendment to the protocol. This was added due to several stakeholder groups and the research literature indicating that measures of intention to leave are paramount to evaluation and implementation of interventions, and especially salient considering the current international healthcare workforce crisis. The questionnaire was distributed via email to *all 62 nurses* who completed the whole programme (thus, both workshops and both coaching calls). A £5 voucher was offered to all as an incentive to participate.

### Analysis plan

#### Quantitative analyses

A more detailed report of the analysis can be found in the study protocol paper [19]. Data were analysed with both R and SPSS. Multilevel (random intercepts for participants) regression models for each outcome included a timepoint coefficient, and were unadjusted, or sequentially adjusted for gender, age, and experience (years in profession). Holm-corrected *t-*tests further assessed between timepoint differences in outcomes.

#### Qualitative analyses

As per protocol, reflexive thematic analysis (RTA) [43] was used to analyse the interviews. RTA does not require a pre-determined ontological or epistemological framework; and is therefore commonly used in applied health research. KSV coded all interviews; and RSE coded a subset of these [*n* = 3, 20%]. Similarities and differences in coding was discussed between the researchers; however, the researchers generally agreed on the use of codes and salient aspects to code.

## Results

### Participant characteristics

A total of 84 participants consented to participate in the study. Most participants were female [86%], and their mean age was 39.7 [SD = 9.2; range: 22-60; missing *n* = 3]. Participants’ years of experience as registered nurses ranged from 0 [i.e., less than 1 year’s experience] to 39, with a mean of 13.9 [SD = 9.0, missing *n* = 3]; while their years of experience as registered nurses in critical care ranged from 0 to 35, with a mean of 10.7 [SD = 8.8, missing *n* = 3]. Three CCNs indicated that they were off work with stress when they completed the baseline questionnaire; however, when/if those nurses return to work was not followed up. At baseline, two CCNs were also taking part in other workplace wellbeing initiatives, and four others indicated that they had taken part in workplace wellbeing initiatives in the past. Fifteen interviews were conducted by KV (24% of participants), one of which was a pilot interview to trial the interview schedule, so is not included in the analysis.

### Intervention delivery

Twenty-five workshop cycles were offered to participants. Nineteen cycles were chosen by participants, 6 cycles were cancelled and participant numbers in each ranged from two to six participants.

### Primary feasibility outcomes

#### Demand

A total of 102 UK CCNs signed-up to the study by booking a place; thus, the target of recruiting at least 80 CCNs was met.

#### Recruitment

A total of 84 CCNs consented to participate in the study. Out of the 84, 77 attended the first workshop [91.7%], thus the objective of recruiting at least 80 CCNs was not met, but this was within 5% of the goal figure.

#### Programme retention: online, remote delivery of reboot

Of the 102 sign-ups, there were 62 completions, 15 dropped-out during the programme and 25 signed up but did not attend or cancelled their first workshop. Out of the 77 who attended the first workshop, 62 completed both workshops and both coaching calls; thus, 80.5% of those who attended the first workshop completed the programme – this means that the objective of achieving a (participation) retention rate of ≥70% was met.

#### Retention: feasibility of evaluation of online, remote delivery of reboot

Out of the 77 who completed the first workshop, 58.4% completed final, time-4 measures. Thus, the objective of ≥50% completion rates for the final follow-up questionnaire was met.

### Secondary outcomes

The *secondary* objective was to provide a preliminary assessment of whether Reboot could potentially significantly increase both self-reported psychological resilience and confidence in coping with adverse events, via analysis of questionnaires and interviews.

### Quantitative results

Descriptive statistics are presented in Tables 1, 2 and 3; and model-fit and results are presented in Tables 4 and 5 (4 for unadjusted models, 5 for adjusted models). All analyses indicated considerable clustering, supporting the use of random intercepts. The proportion of variance explained by all indicator variables was sizeable across measures, however, a higher proportion of variance was explained by fixed time points explained plus random effects. Adjusting the model for gender, age and experience did not alter model fit, thus are not reported here but results can be viewed in Table 4.

**Table 1:** Descriptive statistics for all outcome measures

Measure (*timepoint)*	Mean (SD)	Median	Min, Max	Missing	Missing %
**Confidence in coping with adverse events**					
*T1*	8.51 (1.83)	9	3, 12	0	0
*T2*	11 (1.15)	11.5	9, 12	24	28.57
*T3*	10.9 (1.29)	12	9, 12	45	53.57
*T4*	11.2 (1.25)	12	7, 12	46	54.76
**Knowledge of resilience**					
*T1*	3.27 (1.16)	3	1, 5	2	2.38
*T2*	4.42 (0.979)	4.5	2, 6	24	28.57
**Resilience (BRS)**					
*T1*	18.4 (3.97)	18	6, 27	1	1.19
*T3*	20.8 (3.70)	21	11, 27	45	53.57
*T4*	20.7 (3.65)	21	12, 29	45	53.57
**Burnout (OLBI-abbreviated)**					
*T1*	15.9 (3.07)	16	7, 23	23	27.38
*T3*	13.4 (2.41)	13	6, 18	45	53.57
*T4*	13.4 (2.92)	13	7, 19	46	54.76
**Depression (PHQ-9)**					
*T1*	8.18 (5.12)	7	0, 24	2	2.38
*T3*	3.95 (3.29)	3	0, 16	47	55.95
*T4*	3.77 (4.41)	2	0, 22	45	53.57

**Table 2:** Feedback for the workshops

Item	Strongly Agree	Agree	Neither agree nor disagree	Disagree	Strongly Disagree	Total n
The workshops were relevant to my professional group	49	11	0	0	3	63
I learned skills in the workshops which will be useful in future	41	18	1	1	2	61
There was adequate time to cover the material	41	21	0	0	1	63
I found the workshops engaging	44	18	0	0	1	63

**Table 3:** Evaluation of the workshops

Item	Yes	No	Total n
Were there aspects of the workshops you did not find useful?	6	58	63
Is there anything else you would have liked to see in the workshops which was not included?	6	55	61
If you were involved in a stressful workplace event, would you do anything differently as a result of attending the workshops?	57	4	61

**Table 4:** Unadjusted models: Results

Outcome	BIC	Variance (ICC)	R^2^m	R^2^c	Predictor	Contrast	beta	95% CI
Confidence	327	.40	0.38	0.57	Time	T2 versus T1	0.80***	0.66 - 0.94
						T3 versus T1	0.75***	0.59 - 0.91
						T4 versus T1	0.85***	0.68 - 1.01
Resilience	293	.55	0.10	0.64	Time	T3 versus T1	0.39***	0.23 - 0.56
						T4 versus T1	0.42***	0.26 - 0.59
Burnout	207	0.57	0.13	0.68	Time	T3 versus T1	−0.37***	− 0.50- (− 0.25)
						T4 versus T1	−0.39***	−0.52 – (− 0.26)
Wellbeing (PHQ)	226	0.58	0.16	0.71	Time	T3 versus T1	−0.45***	−0.59 – (− 0.32)
						T4 versus T1	0.48***	−0.6 – (− 0.35)
Knowledge	14	0.22	0.22	0.28	Time	T2 versus T1	0.23***	0.16-0.30

**Table 5:** Adjusted models for experience, age and gender

Outcome	BIC	Variance (ICC)	R^2^m	R^2^c	Predictor	Contrast	beta	95% CI
Confidence	324	0.41	0.38	0.58	Time	T2 versus T1	0.79***	0.64 - 0.93
						T3 versus T1	0.75***	0.58 - 0.92
						T4 versus T1	0.82***	0.65 - 0.99
					Age		0.01	−0.01 - 0.02
					Gender	Male versus Female	−0.07	− 0.33, 0.20
					Experience		−0.01	−0.03, 0.01
Resilience	288	0.53	0.15	0.62	Time	T3 versus T1	0.40***	0.22 - 0.57
						T4 versus T1	0.42***	0.24 - 0.59
					Age		0.02	−0.01 - 0.04
					Gender	Male versus Female	0.23	−0.15- 0.60
					Experience		−0.02	−0.04 - 0.01
Burnout	211	0.57	0.15	0.68	Time	T3 versus T1	−0.36***	−0.49 – (− 0.23)
						T4 versus T1	−0.38***	−0.51 – (− 0.25)
					Age		0.00	−0.02 - 0.02
					Gender	Male versus Female	0.25	−0.56 - 0.06
					Experience		0.00	−0.02 - 0.02
Wellbeing (PHQ)	233	0.58	0.18	0.71	Time	T3 versus T1	−0.46***	−0.60 – (− 0.32)
						T4 versus T1	−0.48***	−0.62 – (− 0.34]
					Age		0.00	−0.02 - 0.02
					Gender	Male versus Female	−0.11	−0.46 - 0.23
					Experience		−0.01	− 0.03- 0.02
Knowledge	9	0.19	24%	27%	Time	T2 versus T1	0.23***	0.16 - 0.31
					Age		0.00	0.00 - 0.01
					Gender	Male versus Female	−0.06	−0.18 - 0.05
					Experience		0.00	−0.01 - 0.01

#### Confidence

Confidence scores increased significantly, compared to pre-intervention (Time 1) [Time 2: unadjusted β = 0.80, CI: 0.66 - 0.94, *p* < .001, d = .81; Time 3: unadjusted R^2^ = 0.75, CI: 0.59 - 0.91, *p* < .001, d = 0.78; Time 4: unadjusted R^2^ = 0.85, CI: 0.68 - 1.01, *p* < .001, d = 0.80]. Post-hoc t-tests comparing timepoint means showed no further increase in confidence when comparing between T2, T3 and T4, indicating that initial increases were maintained and remained stable [range *p*_holm_ .977; adjusted for multiple comparisons]. Cronbach’s α for the confidence measure ranged from 0.64 - 0.87 across timepoints.

#### Knowledge

Knowledge scores increased significantly between Time 1 and Time 2 [unadjusted β = 0.48, CI: 0.16-0.30, *p* < .001, d = 0.79].

Descriptive statistic present sums of items, whereas models used the mean of items.

#### Resilience

Resilience scores increased significantly between Time 1 and Time 3 [unadjusted β = 0.39, CI: 0.23 - 0.56, *p* < .001, d = 0.43] as well as between Time 1 and Time 4 [unadjusted β = 0.42, CI: 0.26 - 0.59, *p* < .001, d = 0.49]. Post-hoc tests comparing timepoint means indicated that there was no further increase in resilience between Time 3 and Time 4 (*p*_holm_ = .75), suggesting that initial gains remained stable. Cronbach’s alpha for the three time points it was used at, was between .80-.83; thus indicating good reliability.

#### Burnout

Burnout scores decreased significantly between Time 1 and Time 3 [unadjusted β = −.037, CI: − 0.50- (− 0.25), *p* < .001, d = − 0.51 and between Time 1 and Time 4 [unadjusted β = −.039, CI-0.52 – (− 0.26), *p* < .001, d = − 0.56]. Post-hoc tests showed no significant difference was found on the BRS between T2 and T4; *p*_holm_ = .82, indicating that decreases were maintained and remained stable. Reliability for the questionnaire was good, with Cronbach’s alpha ranging from .76-.84 for the three time points.

#### Depression

Scores on the PHQ-9 indicated a significant decrease in depression from both Time 1 to Time 3 [unadjusted β = −.045, CI: − 0.59 – - 0.32, *p* < .001 as well as from Time 1 to Time 4 [unadjusted β = −.048, CI: − 0.6 – (− 0.35); *p* < .001].

Post-hoc tests showed no significant difference on PHQ-9 scores between T3 and T4; p_holm_ = .73, indicating that reductions were maintained and remained stable. Cronbach’s alpha for the PHQ-9 for the three time points it was used ranged from .83-.88, thus indicating good reliability. Out of the 39 participants who completed the PHQ-9 at both baseline and Time 4, almost 80% of participants screened for the presence of mild or severe depression at baseline (PHQ-9 score of 4 or above), whereas at Time 4, only 31.8% did.

#### Feedback/reactions

Feedback and reactions to the Reboot workshop were overwhelmingly positive (Tables 2 and 3). Most participants agreed or strongly agreed that it was relevant for their professional group; they learned useful skills and felt the workshops were adequate in length and were engaging. The majority also indicated that they would react differently if they were involved in a stressful workplace event after attending the workshop. Only a minority (*n* = 5) indicated that there were aspects of the workshops that they did not find useful. Seventy-two CCNs answered the question as to whether they would recommend the workshops to other HCPs; 71 indicated that yes, they would, whereas one participant said they would not.

#### Intention to leave

Thirty-two out of the 62 nurses who completed the full programme responded to the invitation to complete an additional questionnaire in May 2022 (response rate: 51.6%). Participants were asked to answer a set of questions measuring their turnover intentions as they recalled them prior to participating in Reboot (pre-Reboot), and as they are now (post-Reboot). Higher scores indicate lower intention to leave. Using a paired-samples t-test, a significant difference in intention to leave between pre-Reboot (mean = 11.50, SD =2.64) to post-Reboot (mean = 13.56, SD = 1.63) was found [t (31) = 4.93, *p* < .001, d = 0.94], showing that nurses reported significantly lower intention to leave critical care nursing after completing the programme than before. Cronbach’s α for post-Reboot was 0.73 and 0.78. for pre-Reboot.

### Qualitative results

**Fig. 1:**
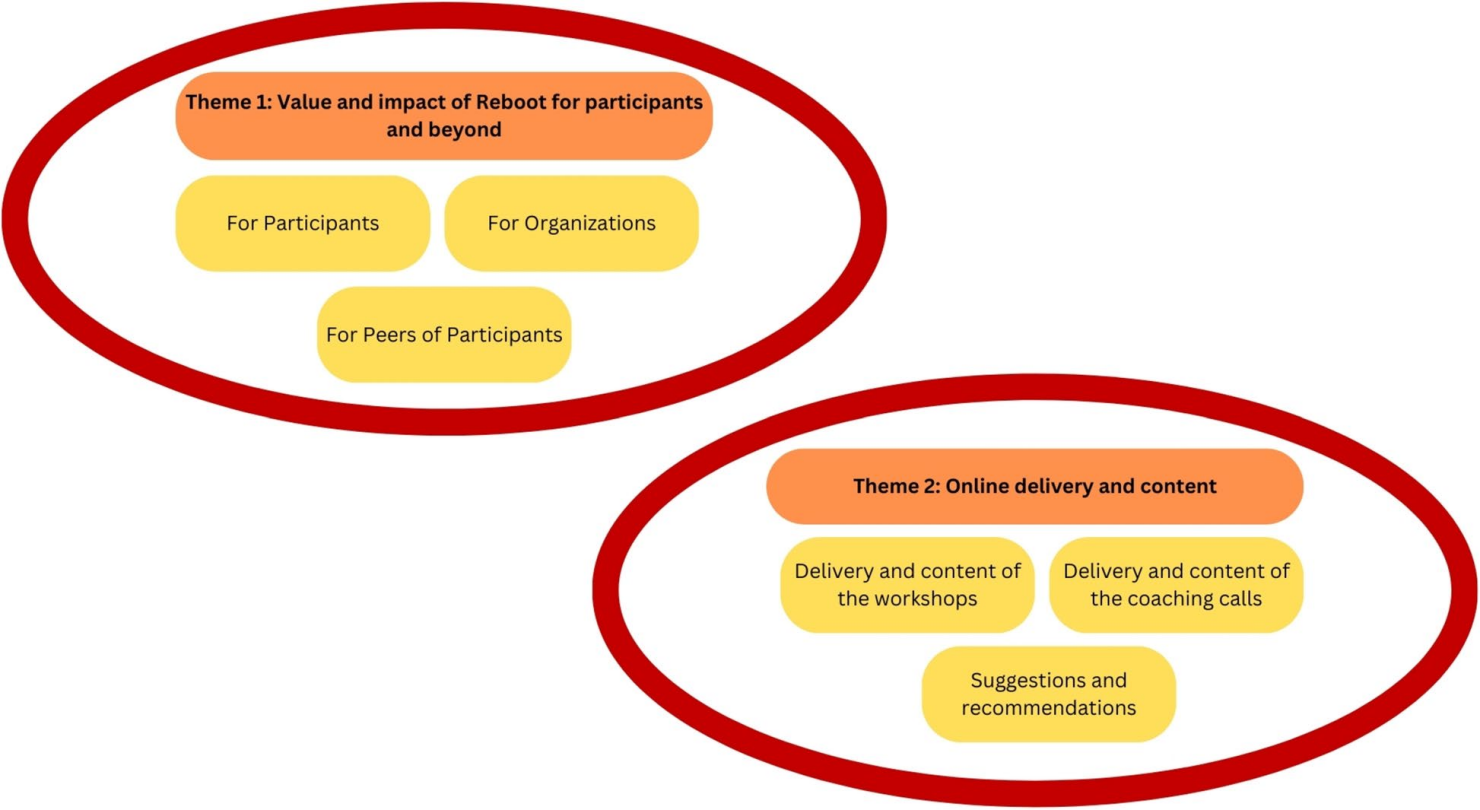
Graphic representation of qualitative findings

From the 15 interviews, two themes were developed. These were: *“The value and impact of Reboot for participants and beyond*” and *“Online delivery and content”.* Both themes had subthemes, illustrated in Fig. 1.

### Theme 1: the value and impact of reboot for participants and beyond

The value and impact of Reboot was described as *“priceless*” (Interview 14) for participants themselves, for their peers with whom they were able to share the psychological tools and knowledge with, and for organisations.

#### The value and impact of reboot for participants

Specific benefits that participants identified for themselves as a result of attending Reboot were better understanding of their own thought processes and emotions, better understanding of why errors happen at work, having a “*tool kit*” (Interview 15) of simple, psychological tools that they can draw on in times of stress (both at work and outside of work), being able to better manage mental and physical stress, and being able to better compartmentalise work and life outside of work.

Participants also specifically identified increases in wellbeing, confidence and knowledge about resilience, and decreases in burnout and intention to leave (Table 6). In addition, CCNs expressed that the group workshops made them feel validated in their feelings of stress, and that it was helpful to meet other professionals outside of their organisation who had similar experiences:

*“… but actually speaking to other people who'd been through a similar experience, who they wish they'd done some things differently as well you know… made you kind of realize we are human, we tried our best and hindsight is a wonderful thing, and experience is a wonderful thing…” (Interview 4)*

**Table 6:** Benefits of Reboot to the Individual (Audit trail)

Concept measured in quantitative outcome measures	Reflected in qualitative interviews
Wellbeing	*“it’s… made me a bit more in tune with myself and sort of learning to deal with the sort of, the emotions of work a bit more, and sort of taking a step back a little bit, and take care of yourself a bit more, you know… so it has - it has definitely, definitely helped.”* “*I’m probably in a better place to be able to do that now because I can recognize it now* ***[after Reboot]…*** *because it.- sometime it’s like, if you’ve got so much negative stuff in there, there’s no room for anything good*”
Burnout	*“I think it’s meant a lot, you know the recognition of the difficult time that critical nurses have been through, and I think you’ve saved a lot of nurses moving away from critical care as well, I really do, because I’ve had a lot of corridor conversations with nurses who, they can’t do it anymore, but having that recognition that it is difficult and that there Is help there for people to carry on yeah I think Is huge. I think the impact has been huge, so thank you.”* *“It’s really important tool to be able to build sort of resilience and manage threats, and because it is such a big problem in the NHS at the moment with my colleagues, there is so much burnout that that I think it’s just all the different techniques and things like that are all really useful - I just think there’s so many people at work that at the moment that I wish could do the course - because I think they’d really benefit from it.”*
Intention to Stay	“*think it’s going to help me stay in the job, and to take- take a step back, take a step back, reflect on things in a neutral kind of way rather than very sort of emotionally charged way, and I’m sure that it will give me the staying power because things are going*.” “*Retention… I mean… ultimately yes because if you improve how your staff feel and how they approach their work, and… feel like they have those tools to cope and, you know, to be resilient in their practice, then you would ultimately, you know, prevent that, you know, prevent dropout and improve - you know, would be say improve attrition rates…? You stop people dropping out.*” “*I think it’s a really important area that I think more nurses should have access to things like this to be able to… carry on doing the job*.” “*I’m sure they [skills gained during Reboot] are useful, because I think it’s going to help me stay in the job, and to take- take a step back, take a step back, reflect on things in a neutral kind of way rather than very sort of emotionally charged way, and I’m sure that it will give me the staying power because things are going - always going to come up at work I think that are challenging.”*
Confidence	*“I went to some training a few, a couple of months ago now, and they were going over, it- it was, it was like a learning thing, but then they went over some mistakes that had been made and near misses and stuff and I came away and I thought I’d come away feeling, really good cause I’d had this training and like I’d feel more knowledgeable, I came away like terrified like oh my God, that’s gonna be me that’s gonna be me doing those mistakes, and they’re gonna be talking about me one day in those- in those and, and I couldn’t even think about it whereas I think, yeah I’m definitely more relaxed about things now, and just more accepting, and yeah just- I just feel calm- like calmer and more relaxed about it yeah. [Interviewer: Lovely.] I don’t- I don’t- I’m not very good at putting this into words, sorry.”*
Knowledge	*“I guess I always thought resilience was being like a really, really strong and been able to bat anything off, but I think it’s more about having coping mechanisms to be able to deal with the things that are thrown at you, rather than being this supersonic person. It’s just been able to have mechanisms to deal with it really.”* *“I had, you know, … everyone has heard about it, haven’t they? They’re all the sort of buzz words isn’t it, resilience, but this is the first time anybody’s actually practically sort of helped me build some resilience.”*

#### The value and impact of reboot for peers of participants

Five participants, who were predominantly senior CCNs, also expressed that after Reboot, they were “*also able now to help other people bounce back* “(Interview 14) by sharing knowledge and tools learnt during Reboot.

*“I was sat with them one of my nurses who, who thought they'd made an error. I'm not sure they did but they were being really hard on themselves, they were ruminating and going over and over and over, to a really unhealthy extent. So, I brought in some of what we've done at the workshop, and I said you know this is what you're doing and it's not healthy and these are ways that you can you know, you need to try and break the cycle.” (Interview 4)*

#### The value and impact of reboot for organisations

Being offered training around psychological tools to cope with stress and how to boost resilience was described as essential by CCNs, as otherwise they would not be able to do their jobs. Thus, CCNs drew links between accessing programs like Reboot and the sustainability of the workforce in critical care.

*“I'm sure that it will give me the staying power because things are going - always going to come up at work I think that are challenging”* (Interview 13)

*“you've got to have a lot of resilience to be able to even want to turn up*” (Interview 2)

All participants said that Reboot should be offered to nurses early on in their nursing career, especially within the first year of working in critical care.

*“everybody else had ought to be going” (Interview 7)*

“I think really early on in their career, to be able… to know how to approach negative thinking habits… to stop the rumination;… the amount of time I have ruminated on situations and blamed myself for things… they really do play on your mind for weeks sometimes. I think having these tools, so just to have them really early on in your career, so you know how to, how to approach those situations…” (Interview 15)

One participant suggested that while the value of Reboot lay in its focus on the acute, stressful situations that occur in intensive care settings, it does not address more long-term problems, such as issues with turnover and short staffing which are also affecting staff wellbeing.

“I think it's a bit more difficult with everything that's happening due to Covid and staffing at the moment with us, because we've got a lot of turnover of staff because, I guess, people just aren’t happy, but in acute situations, definitely.” (Interview 2)

### Theme 2: online delivery and content

This theme incorporates narratives around the delivery and content of the workshops, coaching calls and suggestions for improvement.

#### Delivery and content of the workshops

Participants spoke positively about the online delivery of Reboot. The workshops were perceived as “*delivered at the right pitch” (Interview 14)* and *“comfortable*” (Interview 4), with no problems with internet connectivity. Participants liked the presented background to the interventions, the opportunity to share their experiences with other CCNs and the practical content of the workshops. Some participants commented on the fact that the ‘*homework’* set between the first and second workshop was helpful as it made them more conscious of what they were doing and feeling. Participants liked having the workbook to accompany the sessions, and to have as a point of reference for the future.

‘I liked the activities that you did and it was quite personal to you, so you could bring your own experiences, and use them and we went through them, shared with each person how you could use strategies to help you. That was good as well.” -Interview 2

“Really enjoyed and it was very active, not like one speaker is speaking and someone else just listening in - no, really, everything was really practical, in a realistic way… It was really a natural, realistic knowledgeable feeling.” - Interview 11

#### Delivery and content of the coaching calls

The coaching calls following the workshops were described as deepening understanding, empowering, helpful, professional, and relaxed. Participants spoke very highly of the CBT therapist delivering the intervention, praising their kindness and helpfulness. CCNs felt understood, and appreciated the individualised support, which often included specific materials being sent to them by email following the coaching calls.

*“they [the coaching calls] were probably the most helpful” -Interview 2*

“Touched in every corner… whenever I got a doubt, I was suddenly sharing with [therapist name] and she was listening and giving some kind of tools and… it was really touching it.” – Interview 11

“I thought, I thought she was brilliant… and really kind, and she listened… I was really thankful.” – Interview 13

One of the participants described the coaching calls as “*invaluable*” (Interview 14) and liked the fact that the coaching calls gave her opportunity to discuss what she was struggling with and tools to solve the problems for herself, with support of the therapist, rather than being given a solution to a problem.

*“Quite invaluable and as a supported tool… because it wasn't like, …“this this is the problem… okay well, here's the answer”, it wasn’t that.. it was a “right, well that is the problem, let’s look at some tools you can use to help and support you to find a way through that yourself”, which was really empowering…. It's not sort of… “this the problem I had…this is how you fix it… this is what you've got to do. It wasn't that - it's “here's some tools, work through those tools, see what you think when we come back on the next coaching call” … very empowering, so I had to sit there and do that myself, which was great.”* – Interview 14

#### Suggestions and recommendations

Participants made a small number of suggestions to improve Reboot, which included a slightly longer workbook with more content, to ensure that content is not forgotten about, more coaching calls and delivering some *‘refreshers’* on content later on. One participant also suggested including an example about long-term stressors, such as sustained short staffing.

Unlike with the workshops, participants did not receive reminders about their coaching calls from the research team or therapist, which meant that some participants forgot they had booked their coaching calls. Some participants suggested reminders about upcoming coaching calls would be beneficial, to ensure they remember and attended.

## Discussion

The current study sought to assess the feasibility of delivering Reboot via online, remote delivery to CCNs, and to provide a preliminary assessment of whether Reboot could potentially increase resilience and confidence in coping with adverse events and decrease burnout, depression, and intention to leave. The results suggested that it is feasible to deliver Reboot via online, remote delivery to CCNs, and found significant increases in resilience and confidence in coping with adverse events and decreases in burnout and depression. Retrospective recall also indicated that nurses believed they had reduced intention to leave after participating in the programme. The qualitative findings echoed the quantitative findings, with CCNs particularly valuing the practical exercises that could be translated into everyday practice.

These findings support those of previous studies indicating that Reboot may be a valuable intervention for HCPs [37, 38, 44], but also extend this in four main ways.

First, the current results were the first to indicate that Reboot may have value in a post-pandemic context. Pre-pandemic, there were already around a third of doctors and nurses suffering from burnout and significant increases reported for work-related stress among healthcare staff [37, 45]. However, rates have increased internationally following the onset of the pandemic [46, 47]. In the UK, the General Medical Council (GMC) has been running its annual workforce burnout survey since 2018, making it the largest and most comprehensive annual workforce survey in the UK. In 2022, the burnout risk for doctors was at its highest since 2018. In 2021, 46,793 UK medical trainees completed the survey; 43% said that they found their work emotionally exhausting to a high or very high degree, and 33% indicated that they were feeling burnt out from work to either a high or very high degree [48]. A year later, in 2022, the numbers worsened as 39% (a 6% increase) of trainees indicated that they were feeling burnt out to either a high or very high degree, and 51% of trainees (8% increase) indicated that they found their work emotionally exhausting to a high or very high degree [49]. While, unfortunately, there is no equivalent study of this scale and magnitude for nurses, this survey, alongside reports from the Nursing and Midwifery Council, show just how extreme the situation has become in healthcare in the UK [50], and that HCPs desperately need support. While it was possible that these increases in burnout across healthcare professions may have rendered Reboot unworkable or irrelevant, this study shows that Reboot is still feasible and potentially effective, even in the context of psychological changes within the healthcare workforce.

Second, the current results extend the existing literature by showing that Reboot is feasible and potentially effective for CCNs in particular. To date, there are no systematic reviews or meta-analyses that assess the efficacy of intervention to increase resilience or decrease burnout in CCNs. There are, however, reviews that either assess the efficacy of interventions on reducing burnout, or increasing resilience, in physicians and nurses concomitantly [35, 51, 52], or the efficacy of resilience or interventions more generally [36, 53]. Overall, these reviews conclude that online programmes and internet-based interventions, as well as psychosocial training interventions, are among the interventions that have a positive effect on burnout and resilience, and that CBT-based resilience interventions and mixed-methods most effective at increasing resilience [36, 51, 53]. However, one major criticism of existing interventions is that they are generic and lack relevance for the work stresses different types of HCPs, or even nurses, are facing. Reboot overcomes this by the fact that it can be tailored to each disciplinary group, including critical care nurses or other specialist areas of nursing, ensuring relevance and saliency of the material for specific discipline groups, rather than for HCPs more generally. For example, CCNs will require different content to be included in a resilience and burnout intervention that is salient and acceptable to them, compared to trainee doctors, surgeons or midwives [37] but also compared to other nurses. CCNs tend to have different psychological profiles, compared to non-CCNs. For example, nurses working on either orthopaedic or dialysis wards have been found to have much lower burnout scores, compared to nurses working on critical care units [54] – a difference that has likely been further exacerbated by the pandemic, and effective resilience interventions must take this into account. This will also be a challenge for implementation into practice, as delivery of Reboot would need to be planned and tailored in advance for each HCP group.

Third, the current results also add to the wealth of evidence for the efficacy of person-directed interventions [36, 51]. Person-directed interventions can be defined as those which aim to improve an individual’s capacity to cope with the demands of their job, which is often achieved via mindfulness or CBT programmes. While the quantitative findings highlight that Reboot is feasible and potentially effective for CCNs, the qualitative findings add important knowledge to the aspects of person-directed interventions which CCNs found valuable. For example, participants especially valued the practical applications of the programme which helped them, and by proxy, their peers, cope with the demands of their critical care nursing. In this context, it is not possible to suggest that Reboot can be considered superior to other existing interventions for nurses but as one of several candidate interventions which should be tested using more rigorous research designs. However, it should be noted that Reboot has some unique features not shared with other existing interventions: for example, it involves a mixed-modality format, ensuring the benefit of both peer support and one-to-one confidential space with a therapist.

It is clear that interventions, such as Reboot, cannot compensate for organisational failings; and should be used alongside, rather than in place of, organisational interventions [19, 37, 55]. However, organisational changes are often decided at a regional or national level and influenced by political and economic factors. As such they can be challenging to implement. In this context, person-directed interventions are often appealing to organisations as they are within their decision-making latitude/capability to select and deliver. At the same time though, staff often do not currently have the time to attend, and engage with, wellbeing programmes on offer, leading to lack of uptake and furthering intention to leave among NHS employees [56]. Thus, organisational changes that allow the attendance of, and engagement with, wellbeing programs are desperately needed, alongside changes that have been associated with reduced burnout in nursing, such as higher pay, more work flexibility, higher autonomy and fewer/better working hours [57–60].

Fourth, the present study also contributes to a growing literature which is focused on the prevention rather than amelioration of work-related mental distress. Research is starting to highlight the importance of higher levels of resilience as protective factors against burnout and the development of post-traumatic-stress disorder (PTSD) for CCNs, and beyond [30, 36, 61–63]. For example, a 2021 study conducted in Poland [63] found that higher levels of resilience were associated with lower levels of burnout and secondary traumatic stress, while exposure to secondary traumatic stress was positively related to burnout. This supports the development, and implementation, of prophylactic resilience interventions for healthcare staff, rather than ameliorative burnout or PTSD interventions.

## Limitations

While strengths of the current study include its mixed-methods design, which can elucidate not just the potential impact of the intervention but also the mechanisms underlying this, there are a number of limitations.

Firstly, the current uncontrolled study design means that causal associations between Reboot and the outcomes measured cannot be assumed. Higher quality evidence, perhaps in the form of a wait-list control design or randomised controlled trial, is now needed.

Secondly, intention to leave scores were collected retrospectively for pre- and post-Reboot, thus future work should include intention to leave measures from baseline.

Thirdly, the majority of workshop and coaching sessions were delivered by the same therapist, a future trial should ensure the inclusion of multiple therapists. The therapist also encouraged the completion of outcome measures (especially at Time 2, post completion of second workshop), which means the therapist was not entirely independent of the evaluation.

Fourth, non-completers were not further followed up or invited to interview. Future research should consider their perspectives too.

Fifth, due to the study focus on CCNs, generalization to nurses working outside of critical care is not possible.

## Conclusion

The current results suggest that it is feasible to deliver Reboot via online delivery to CCNs, and that it is associated with self-reported increases in resilience and confidence in coping with adverse events and decreases in burnout and depression. Participants also reported that their intention to leave reduced following the programme. The qualitative findings echoed the quantitative findings, with CCNs particularly valuing the practical exercises that could be translated into everyday practice. These findings, alongside those of the previously investigated in-person (rather than remote) version support the evidence-base and efficacy of Reboot. However, a randomised controlled trial design is now needed to more fully and robustly ascertain the efficacy of Reboot.

### Supplementary Information

**Additional file 1.**

## Data Availability

Anonymised behavioural data and statistical analysis may be requested via email from Dr. KS Vogt, after data collection and publication of results.

## Abbreviations

BRS: Brief Resilience Scale
CBT: Cognitive Behavioural Therapy
CCN(s): Critical Care Nurse(s)
HCP(s): Healthcare professional(s)
NHS: UK National Health Service
PHQ-9: Patient Health Questionnaire
PTSD: Post-traumatic stress disorder
Reboot: Recovery boosting coaching programme
SRF: Senior Research Fellow
UK: United Kingdom
