# Supplementary material for: Can the Reboot coaching programme support critical care nurses in coping with stressful clinical events? A mixed-methods evaluation assessing resilience, burnout, depression and turnover intentions
Source: BMC Health Serv Res. 2024 Mar 15;24:343. doi: 10.1186/s12913-023-10468-w (PMC10941361; doi:10.1186/s12913-023-10468-w)
Supplement: Supplementary file 1 — Additional file 1. [file 12913_2023_10468_MOESM1_ESM.docx]

**Appendix 1: Interview Schedule/Topic Guide**

**Topic Guide - Follow-up Interviews with Participants**

*Outline of the project. Check participants’ understanding of the study information, offer an opportunity for additional questions and go through the consent process.*

*I’d like to start quite broadly really – Could you tell me a little bit about yourself, please, and how you came to do Reboot.*

*What was your overall experience of the programme?*

**RESILIENCE and ERROR MANAGEMENT**

1. Can you describe to me your understanding of the concept of resilience?
   1. Had you come across this concept prior to the workshops?
   2. Do you feel that you have a good understanding of the concept after taking part in the workshops?
2. To what extent do you feel that higher levels of resilience may help you in your professional practice?
3. Since engaging in the workshop, how do you feel about your ability to cope with instances of error arising during your work?
4. To what extent do you think that being more resilient may help you to manage instances in which you have made a mistake in the course of your work?
   1. In what ways if any do you think that being more resilient may help you?
   2. Do you feel that the workshop has provided you with any useful skills for such instances?
5. To what extent do you feel that the skills developed are relevant and useful to your future career as a health professional?

**WORKSHOP and follow up phone-call/tutorial**

I’d now like to ask you a bit about you’re your experience of the workshop.

1. What was your overall perception of the workshop?
   1. Probe particular issues arising here
2. What did you think worked well?
3. What could be improved?
   1. Probe as to how improvements might be made
4. What was your experience of the follow up phone-call/tutorial?
   1. Probe particular issues arising here
5. How did this call contribute to your learning and overall professional development in relation to this topic?
   1. Probe particular issues arising here
6. Overall would you recommend this session to others undertaking your professional training?

*That brings us to the end of the question I have for you but do you have anything you would like to add or any questions for us? Thank the participant.*
